# Low muscle strength and increased arterial stiffness go hand in hand

**DOI:** 10.1038/s41598-021-81084-z

**Published:** 2021-02-03

**Authors:** Maximilian König, Nikolaus Buchmann, Ute Seeland, Dominik Spira, Elisabeth Steinhagen-Thiessen, Ilja Demuth

**Affiliations:** 1 Department of Endocrinology and Metabolism, Charité-Universitätsmedizin Berlin, Charitéplatz 1, 10117 Berlin, Germany; 2 Department of Cardiology (Campus Benjamin Franklin), Charité-Universitätsmedizin Berlin, Hindenburgdamm 30, 12203 Berlin, Germany; 3 Institute of Gender in Medicine (GiM), Center for Cardiovascular Research (CCR), Charité-Universitätsmedizin Berlin, Berlin, Germany; 4 DZHK (German Centre for Cardiovascular Research), Berlin, Germany; 5 BCRT - Berlin Institute of Health Center for Regenerative Therapies, Berlin, Germany

**Keywords:** Cardiovascular biology, Epidemiology, Biomarkers, Geriatrics

## Abstract

Low handgrip strength and increased arterial stiffness are both associated with poor health outcomes, but evidence on the relationship between handgrip strength and arterial stiffness is limited. In this cross-sectional analysis of combined baseline datasets from the LipidCardio and Berlin Aging Study II cohorts we aimed to examine whether handgrip strength (HGS) is associated with arterial stiffness. 1511 participants with a median age of 68.56 (IQR 63.13–73.08) years were included. Arterial stiffness was assessed by aortal pulse wave velocity (PWV) with the Mobil-O-Graph device. Handgrip strength was assessed with a handheld dynamometer.

The mean HGS was 39.05 ± 9.07 kg in men and 26.20 ± 7.47 kg in women. According to multivariable linear regression analysis per 5 kg decrease in handgrip strength there was a mean increase in PWV of 0.08 m/s after adjustment for the confounders age, sex, coronary artery disease, systolic blood pressure, body mass index, cohort, and smoking. Thus, there was evidence that low handgrip strength and increased arterial stiffness go hand in hand. Arterial stiffness can possibly create the missing link between low handgrip strength and increased cardiovascular morbidity and mortality. Causality and direction of causality remain to be determined.

## Introduction

Understanding the relationship between aging and cardiovascular disease (CVD) is becoming increasingly important in the light of aging populations around the world, leading to a steep increase in age-related morbidity^1^. Aging occurs at varying rates and is a heterogeneous process^2^. Some individuals exhibit typical age-associated conditions and deficits early, whereas others never develop certain symptoms and markers of aging. There may be a discrepancy between an individual’s chronological and biological ages^3^. An individual’s biological age can be estimated using biomarkers. Handgrip strength, as a surrogate measure of overall muscular strength, is an attractive candidate biomarker of aging since handgrip strength is easily accessible and measuring grip strength is simple and inexpensive.

Low handgrip strength is associated with a whole range of poor health outcomes, including incident CVD, and major adverse cardiovascular events (MACE), but also frailty, impaired quality of life, longer hospital stays, disability, cognitive impairment, decrease in renal function, respiratory and cancer outcomes, and premature mortality, respectively^4–10^.

The mechanisms underlying the association between handgrip strength and cardiovascular disease, among others, remain unclear. Although not immediately obvious, skeletal muscle and the vascular system are closely connected; a bi-directional relationship can be assumed^11^. Skeletal muscle has broad physiological and functional roles. While the obvious role is to enable body movements, skeletal muscle is also the primary location of protein storage within the body, the primary glucose consumer, and important in metabolic conditions such as diabetes^12^. Therefore, healthy muscles require, among other things, an adequate supply of nutrients. There is convincing evidence demonstrating an association between vascular function and skeletal muscle health^13^. Both quantitative and qualitative changes in the microvasculature may contribute to reduced muscle mass and function. E.g. it has been suggested that endothelial dysfunction may result in reduced flow due to microvascular dysfunction, and reduced microcirculation may lead to muscle fiber atrophy^14^.

Furthermore accumulating evidence supports coronary microvascular dysfunction as an important component of the explanation for myocardial ischemia and its relation to adverse outcomes^15^. By analogy, it would be conceivable that microvascular dysfunction also plays a major role in skeletal muscular dysfunction (reduced strength and loss of skeletal muscle mass, altered metabolism, e.g. development of insulin resistance)^16,17^.

Recently, some studies suggested that endothelial dysfunction and arterial stiffness might mediate the association between muscle strength and cardiovascular events^18^. Arterial stiffening—i.e. the loss of the arteries’ or the aorta’s elastic properties—is a feature of physiological vascular aging, but may be accelerated in a variety of pathological conditions^19^.

Arterial stiffness can be assessed by measurement of pulse wave velocity (PWV), being considered the gold standard parameter of non-invasively measured arterial stiffness^5,20^. Numerous publications including meta-analyses and systematic reviews have shown associations of pathological arterial stiffness with aging, cognitive decline cardiovascular disease, and traditional and novel CV risk factors^17,21–25^. As with handgrip strength, PWV is highly predictive for cardiovascular events and all-cause mortality^24,26,27^.

The current evidence regarding the association between pulse wave velocity or arterial stiffness and muscular strength, respectively is scarce and conflicting: recently the Wakayama Study showed in community-dwelling older adults (72 ± 5 years) without manifest cardiovascular disease that HGS progressively decreased with an increase in brachial-ankle PWV (baPWV) level^7^.

Another study performed in 1002 Chinese community dwelling adults aged ≥ 65 years showed that PWV was significantly associated with handgrip strength, but only in men, while another study was not able to replicate the finding of handgrip strength being associated with PWV in hypertensive patients^28,29^.

Since evidence on the relationship between handgrip strength and arterial stiffness is limited and in parts inconclusive, the aim of the present study was to investigate the relationship between handgrip strength and arterial stiffness, both in a cardiovascular high-risk sample of patients and in an above-average healthy sample of community-dwelling adults. We hypothesized that lower handgrip strength would be associated with higher arterial stiffness.

## Methods

### Population and design

A cross-sectional study was conducted. We combined baseline data from two cohort studies, the LipidCardio and the Berlin Aging Study II (BASE-II).

The LipidCardio cohort is a cohort of 1005 consecutive patients (70.9 ± 11.1 years) recruited on the occasion of elective coronary angiography at a single large academic center (Department of Cardiology, Campus Benjamin Franklin, Charité-Universitätsmedizin Berlin) during 2016–2018^30^. Patients aged 18 years and older undergoing cardiac catheterization due to (suspected) chronic coronary syndromes, except those with troponin-positive acute coronary syndromes (ACS), were eligible for inclusion. Participation rate was particularly high (> 95%).

All participants gave written informed consent at the time of enrolment. Patients unable to provide informed consent were excluded from the study. The study was approved by the ethics committee at Charité-Universitätsmedizin Berlin (approval number: EA1/135/16).

The population-based sample of the Berlin Aging Study II (BASE-II) has been described previously in detail^31,32^. Briefly, BASE-II was recruited as a convenience sample from the greater Berlin metropolitan area. In 2009–2014, 2171 participants (~ 75% aged 60–85 years and ~ 25% aged 20–35 years) were enrolled in the medical part of the study. All participants gave written informed consent, and the study was approved by the Ethics Committee at Charité-Universitätsmedizin Berlin (EA2/029/09). Of course, generalizability was an issue, which has been dealt with in depth elsewhere^33^.

Both studies were performed in compliance with the World Medical Association Declaration of Helsinki on Ethical Principles for Medical Research Involving Human Subjects, and the manuscript was prepared in compliance with the STrenghtening the Reporting of Observational studies in Epidemiology (STROBE)-Statement^34^.

The LipidCardio study was implemented by the same team of researchers, who had implemented the BASE-II study before. Both studies used the same standard operating procedures (SOP), instruments, and questionnaires, thus reliability within the pooled data set was given.

In both cohorts only in random subsamples PWV measurements had been performed (LipidCardio N = 598 out of total 1005, and BASE-II N = 913 out of total 2171), resulting in a sample size of 1511.

### Pulse wave velocity (PWV)

PWV is generally accepted as the most simple, non-invasive, robust, and reproducible method to assess arterial function^35^. It is defined as the time difference between the start of the forward - pulse wave and the beginning of the reflected wave. PWV in m/s was measured with the Mobil-O-Graph, I.E.M. Germany, a non-invasive oscillometric device, by trained study personnel and according to manufacturer’s instructions. Measurements were performed in a sitting position and relaxed atmosphere^36^. With an upper arm pressure sleeve and oscillometric recording of the A. brachialis waveform and pulse pressure arterial stiffness indices were measured shape and amplitude of central PWV and central (aortic) blood pressure were reconstructed via a mathematical transfer function. For data quality assurance only measurements with good or very good quality were used based on the standard deviation of pulse wave recording for a period of 8 s. PWV was used as a continuous variable in most analyses, and also recoded into a binary variable using 11 m/s as cutoff, which corresponded to the fifth quintile. An accepted threshold for end-organ damage is 10–12 m/s^37^.

### Handgrip strength

Handgrip strength was measured by trained study personnel with a Smedley dynamometer, according to a standard operating procedure (participants either standing or seated, the elbow by their side and flexed to right angles, and a neutral wrist position). Three measurements were made from both hands of each participant. We used only the maximum value obtained from any hand (maximum handgrip strength). Tertiles of HGS were obtained separately for both sexes and low handgrip strength was defined as handgrip below the lowest tertile.

Furthermore HGS as the main exposure variable was divided into 5 kg intervals, and also was recoded into a binary variable (probable sarcopenic vs. normal) according to the EWGSOP2 sarcopenia cut-off points^38,39^.

### Covariables

Blood pressure was measured with an Omron oszillometric device on both arms. For assessment of confounding and effect modification blood pressure was categorized into 5 levels (lowest to 99 mmHg, 100–129 mmHg, 130–149 mmHg, 150–170 mmHg, and greater than 170 mmHg), and age was categorized into 20–40 years, 40–60 years, 60–70 years, 70–80 years, and 80–100 years.

Height and weight were either measured (in BASE-II) or taken over from participants’ records (in LipidCardio). Body mass index (BMI) was calculated as weight in kilograms divided by the square of the height in meters and categorized into < 25 kg/m^2^, 25–30 kg/m^2^, and > 30 kg/m^2^; waist and hip circumferences were measured in light clothes.

Information on current smoking status was sampled during structured interviews, and coded into yes/no.

Classification of coronary artery disease status was based on an up-to-date coronary angiogram in the LipidCardio study, while in BASE-II it was based on self-reported history of CAD or review of medical records provided by the participants.

### Statistics

There were only a few missing values in the combined dataset (less than 5% of the total number of cases per variable).

The outcome of interest was pulse wave velocity (PWV). We treated PWV mainly as a continuous variable, and for some analyses also as binary variable (increased vs. normal PWV). The exposure of interest was handgrip strength, Firstly, we treated grip strength as a continuous variable and calculated coefficients for 5 kg change (increase or decrease) in hand grip strength. We also calculated coefficients for sex specific tertile cut-off points of grip strength in order to facilitate comparability with previous studies, which have used these categories.

Moreover, we did analyses using a sex specific HGS cut-off point for probable sarcopenia as recommended by the EWGSOP2 sarcopenia guidelines^38,39^.

Descriptive statistics were used to describe the pooled study population. Univariable analyses were conducted using all data available. Associations of variables with the outcome of interest (PWV) were examined using linear regression (Table 2), and associations with the main exposure of interest (low handgrip) were calculated using cross-tabulations and crude odds ratios (Table 3), and the chi square test of independence was used to assess statistical significance.

All covariables were tested for confounding and effect modification by calculating stratum-specific estimates for each level of the potential confounder and comparing Mantel-Haenzsel odds ratios to the crude odds ratio.

We computed multivariable linear regression analyses to further examine the relationship between handgrip strength with pulse wave velocity taking confounding into account. We calculated multivariable-adjusted coefficients (ß) for 5 kg increase in handgrip strength including all above-mentioned covariables.

We also performed stratified analyses (by sex, age-group, and cohort) and used the likelihood ratio test (LRT) for interaction to test for evidence of interaction between the main exposure of interest and sex, age, and cohort.

## Results

**Figure 1:**
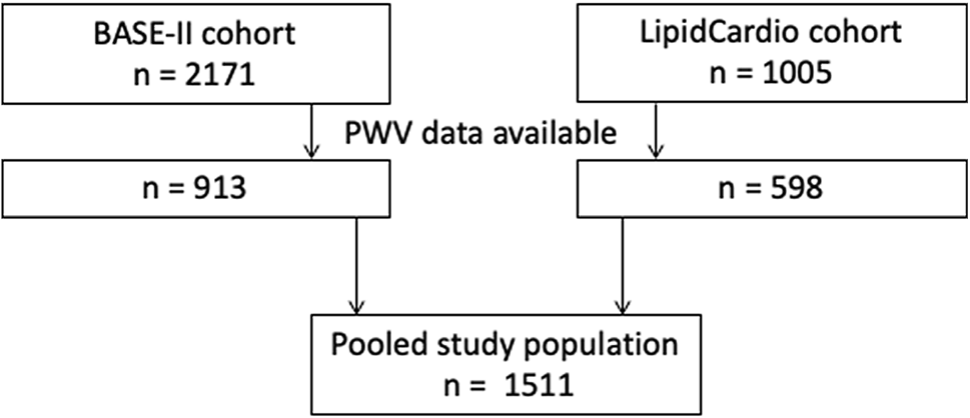
Flow chart of sample selection. In both cohorts only in random subsamples pulse wave velocity (PWV) measurements had been performed (LipidCardio N = 598 out of total 1005, and BASE-II N = 913 out of total 2171), resulting in a sample size of 1511.

Altogether, 1511 participants from the LipidCardio Study (n = 598) and the Berlin Aging Study II (BASE-II, n = 913), who had HGS and PWV measurements at baseline, have been included in this cross-sectional analysis (Fig. 1).

The median age was 68.56 (interquartile range, IQR 63.13–73.08 years. 954 (63.1%) participants were men.

The mean handgrip strength in men was 39.1 ± 9.1 kg, and one third had a handgrip strength of less than 35 kg. In women, the mean handgrip strength was 26.2 ± 7.5 kg, and one third had a HGS lower than 23 kg. Table 1 shows participant characteristics according to sex and handgrip strength tertiles. Participants with low HGS (below the first tertile) were significantly older than participants in the middle group and those with high HGS. They were less likely current smokers and had the highest prevalence of CAD. They had a higher blood pressure than participants in the other two tertile groups; BMI was similar. Not least, those with low HGS showed the highest PWV.

**Table 1:** Participant characteristics by sex and tertiles of handgrip strength.

Total (n = 1511)	Men (n = 954)			Women (n = 557)		
Tertiles	1st (n = 325)	2nd (n = 313)	3rd (n = 316)	1st (n = 198)	2nd (n = 177)	3rd (n = 182)
HGS	29.33 ± 5.25	39.31 ± 2.30	48.78 ± 4.80	18.45 ± 4.08	26.63 ± 1.58	34.12 ± 4.63
Age	71.57 ± 13.04	65.45 ± 12.97	59.34 ± 15.40	72.37 ± 9.21	62.41 ± 15.01	50.93 ± 19.34
PWV	10.47 ± 2.03	9.56 ± 1.78	8.79 ± 1.94	10.53 ± 1.62	9.23 ± 2.08	7.79 ± 2.41
Smoking	46 (14.2)	63 (20.1)	62 (19.6)	24 (12.1)	34 (19.2)	45 (24.6)
BMI	27.11 ± 4.36	26.97 ± 4.38	27.42 ± 3.91	27.11 ± 4.91	25.64 ± 4.74	25.81 ± 5.91
CAD	219 (67.4)	103 (32.9)	71 (22.5)	96 (48.5)	19 (10.7)	12 (6.52)
SBP	136.3 ± 21.9	134.9 ± 17.9	134.8 ± 16.3	134.7 ± 18.7	132.5 ± 19.7	125.8 ± 16.5

Numbers are mean ± SD or number (%); *HGS* handgrip strength, *PWV* pulse wave velocity, *CAD* coronary artery disease, *BMI* body mass index, *SBP* systolic blood pressure.

HGS and PWV showed a moderate negative correlation both in men (r =  − 0.405, *p* < 0.001) and women (r = − 0.499, *p* < 0.001). According to linear regression analysis, per 5 kg decrease in handgrip strength PWV increased on average by 0.30 m/s (ß =  − 0.30, 95% CI  − 0.35 to  − 0.25, *p* < 0.001, Table 2), or by about 1 m/s per tertile decrease of handgrip strength, which is also evident from Table 1.

**Table 2:** Univariate linear regression coefficients of handgrip strength and other covariables with pulse wave velocity.

Characteristic	ß	SE	*p* value
Age groups	0.21	0.003	< 0.001
Female sex	− 0.40	0.115	0.001
Handgrip strength, 5 kg intervals	− 0.30	0.025	< 0.001
Handgrip strength, tertiles	− 1.04	0.062	< 0.001
Low handgrip strength*	1.57	0.110	< 0.001
Probable sarcopenia**	2.06	0.019	< 0.001
Body mass index categories	0.57	0.740	< 0.001
Systolic blood pressure categories	0.20	0.017	< 0.001
Coronary artery disease	1.48	0.112	< 0.001
Smoking	− 1.54	0.139	< 0.001
Cohort (BASE-II vs. LipidCardio)	− 1.30	0.109	< 0.001

ß = regression coefficient, *SE* standard error, *BASE-II* Berlin Aging Study II, *below lowest tertile versus rest, **according to the EWGSOP2 sarcopenia cut-off points.

133 (8.8%) participants were *probable sarcopenic* according to the EWGSOP2 HGS cut-off points^39^. The prevalence of probable sarcopenia increased stepwise with increasing age (< 60 years: 2.0%, 60–70 years: 3.8%, 70–80 years: 8.3%, > 80 years: 47.8%).

Unadjusted, probable sarcopenic participants had on average a 2.06 m/s higher PWV than non-sarcopenic individuals (ß = 2.06, 95% CI 1.69—2.43, *p* < 0.001).

In addition to the association between handgrip strength and PWV the univariable analyses performed showed that PWV was positively associated with age, male sex, body mass index, systolic blood pressure (SBP) and diagnosis of CAD (Table 2), while low HGS was significantly associated with (increasing) age, being overweight, high SBP and a CAD diagnosis (Table 3).

**Table 3:** Characteristics of participants and associations with low HGS (lower tertile).

Characteristic	Category	Odds ratio (95% CI)	*p* value
Sex	Male	1 (Ref.)	–
	Female	1.06 (0.85–1.32)	0.593
Age, years	20–40	1 (Ref.)	–
	40–60	2.98 (1.54–5.77)	< 0.001
	60–70	2.89 (1.76–4.74)	< 0.001
	70–80	7.24 (4.32–12.11)	< 0.001
	80–100	57.69 (20.43–162.86)	< 0.001
BMI, kg/m2	< 25	1 (Ref.)	–
	25–30	1.28 (1.01–1.63)	0.041
	> 30	1.30 (0.97–1.75)	0.078
SBP, mmHg	min-100	2.57 (1.19–5.52)	0.012
	100–130	1 (Ref.)	–
	130–150	1.64 (0.91–1.48)	0.218
	150–170	1.40 (1.02–1.92)	0.039
	> 170	2.22 (1.32–3.75)	0.002
CAD	No	1 (Ref.)	–
	Yes	5.80 (4.50–7.47)	< 0.001
PWV	Low	1 (Ref.)	–
	High	6.12 (4.56–8.21)	< 0.001
Smoking	No	1 (Ref.)	
	Yes	0.59 (0.44–0.80)	< 0.001

*HGS* handgrip strength, *PWV* pulse wave velocity, *CAD* coronary artery disease, *BMI* body mass index, *SBP* systolic blood pressure.

The results of the multivariable analyses of the association between HGS and PWV are shown in Table 4. Controlling for age, sex, BMI, smoking, CAD, blood pressure, and cohort, the association between HGS and pulse wave velocity was attenuated, but still per 5 kg decrease in handgrip strength there was a mean increase in PWV of 0.08 m/s. Given a mean difference of 18 kg between participants in the lowest and the highest HGS tertile this makes a clinically relevant difference of 1.31 m/s in PWV (Table 4).

**Table 4:** Multivariable-adjusted coefficients (ß) for 5 kg increase in handgrip strength.

	ß (95% confidence interval)	*p* value	Number of observations
Overall	− 0.08 (−0.095, −0.054)	< 0.001	N = 1511
**Age groups**			
< 60	− 0.04 (−0.08 to −0.01)	0.026	N = 304
60–70	− 0.07 (−0.11 to −0.33)	< 0.001	N = 581
70–80	− 0.08 (−0.12 to −0.04)	< 0.001	N = 492
> 80	− 0.14 (−0.29 to 0.01)	0.065	N = 134
**Sex**			
Male	− 0.09 (−0.08 to −0.01)	< 0.001	N = 954
Female	− 0.04 (−0.11 to −0.06)	0.093	N = 557
**Cohort**			
BASE-II	− 0.06 (−0.11 to −0.02)	0.002	N = 913
LipidCardio	− 0.09 (−0.14 to −0.05)	< 0.001	N = 598

The effect did not differ significantly by age-group, even if the data showed a trend towards an increasing effect with increasing age. The effect was more pronounced in men compared to women (Table 4).

Moreover, when we examined the relationship between HGS and PWV in the two genuine cohorts separately, the univariate analyses provided consistent evidence that PWV increased with decreasing handgrip strength (LipidCardio: ß =  − 0.33, 95% CI  − 0.39 to  − 0.26; BASE-II:  − 0.16, 95% CI  − 0.23 to  − 0.09), and also after controlling for important confounders there was evidence of an independent association between handgrip strength and pulse wave velocity in both cohorts (BASE-II: ß =  − 0.06, 95% CI  − 0.11 to  − 0.02, *p* = 0.002, LipidCardio: ß =  − 0.09, 95% CI  − 0.14 to  − 0.05, *p* < 0.001; Table 4).

## Discussion

The principal finding of this study was that low handgrip strength (HGS) was associated with increased pulse wave velocity, i.e. arterial stiffness, across a wide age range, and irrespective of age, sex, and cardiovascular comorbidity.

It has been shown before that increased PWV is predictive for adverse (cardiovascular) outcomes^40^. The observed 1.31 m/s difference in PWV between individuals with low and high handgrip strength can be translated into a more than 15% increased risk of CV events, more than 15% higher CV mortality, and more than 15% increased all-cause mortality, which underlines that the relationships investigated here are clinically relevant^41^.

As to the explicit association between pulse wave velocity or arterial stiffness and muscular strength there has been only scarce and conflicting evidence so far: Consistent with our findings, a recent analysis of the Wakayama Study showed in community-dwelling older adults (72 ± 5 years) without manifest cardiovascular disease that HGS progressively decreased with an increase in brachial-ankle PWV (baPWV) level^7^. They could also show that baPWV was significantly and independently associated with appendicular skeletal mass.

Another study performed in 1002 Chinese community dwelling adults aged ≥ 65 years showed that baPWV was significantly associated with handgrip strength, but only in men (OR 1.23, 95% CI 1.07–1.42, *p* < 0.01), whereas there was only weak evidence in women (*p* = 0.07)^28^.

Accordingly, although we were able to show that the association existed consistently in both sexes, it was much less pronounced in women than in men (Table 4).

In contrast, a small study from Brazil was not able to replicate the finding of handgrip strength being associated with PWV in hypertensive patients^29^.

Of note, we could show the association between handgrip strength and pulse wave velocity in both genuine cohorts, in BASE-II, which is convenience sample of community-dwelling older adults whose participants were positively selected regarding health and education, and in LipidCardio, a cohort of patients enriched with manifest cardiovascular disease^30,31^.

Furthermore, our observation is coherent with findings from a steadily growing body of evidence linking low handgrip strength with manifest and preclinical CVD, incident cardiovascular events, cardiovascular mortality, and all-cause mortality, among others, as well as some few studies suggesting an association between low handgrip strength and endothelial dysfunction^42–44^. Of note, these associations have been shown consistently in diverse populations—in the general population, as well as in patients with chronic kidney disease, hypertensive individuals, diabetics, elderly individuals, individuals with CV-disease—and across ethnies^24,42,44,45^.

Likewise, by analogy, several studies have established links between sarcopenia on the one hand and CVD, manifest or preclinical atherosclerosis, and arterial stiffness on the other hand^45,46^.

Yet, low handgrip strength should not be equated with sarcopenia. Individuals with low muscle strength can be regarded as probable cases of sarcopenia. According to the operational definition diagnosis of sarcopenia has to be confirmed by additional documentation of low muscle quantity or quality^38,39^. The EWGSOP2 sarcopenia cut-off points for low strength by grip strength are < 27 kg in men and < 16 kg in women^39^. Sarcopenia is highly prevalent in older adults general population. In this pooled data set 9.0% of men and 8.4% of women (total 8.8%, n = 133) were *probable sarcopenic* cases according to the EWGSOP2 sarcopenia cut-off points. Thus the majority of participants in the lowest tertile group (men: ≤ 35 kg, women: ≤ 23 kg, n = 523) were not *probable sarcopenic*. Indeed, in the present study *probable sarcopenic* participants had on average a 2.06 m/s higher PWV than *non-sarcopenic* individuals according to crude analyses (ß = 2.06, 95% CI 1.69—2.43, p < 0.001), and 0.38 m/s controlling for sex, age, BMI, smoking, SBP, CAD, and cohort (ß = 0.38, 95% CI 0.26–0.50).

What remains to be elucidated is the question of causality and the involved pathophysiological mechanisms. There are a number of puzzle pieces already available: Low muscle strength, muscle mass reduction, and sarcopenia on the one hand, and arterial stiffness on the other hand share some common assumed underlying pathomechanisms that include alterations in endocrine, metabolic, inflammatory, and immunologic systems^47,48^. Common pathophysiologic changes including elevated levels of C-reactive protein, interleukin-6, D-dimer, factor VIII, and insulin resistance have been subsumed as inflammaging, and may be involved in the development of clinical syndromes such as sarcopenia and the frailty phenotype, alterations in markers of CVD such as arterial stiffness and finally CVD and CV events itself^24,49,50^. For example, an analysis from the English Longitudinal Study of Ageing (ELSA) found that higher handgrip strength is associated with lower levels of inflammation at 8-year follow-up, and inflammatory markers partly explained the association between handgrip strength and mortality^51,52^.

Likewise, circulating markers of oxidative stress have been shown to be independently associated with both handgrip strength and sarcopenia, and related to CVD risk^53,54^.

The present study adds to the existing evidence, suggesting that arterial stiffness might be an important intermediate step between muscle strength and CV disease and mortality.

Still, reverse causation is not ruled out. Indeed, it would be conceivable that those with low handgrip strength have subclinical, incipient cardiovascular disease responsible for the lower muscle strength and for the seemingly prospective relation of HGS with CV disease and mortality.

As is commonly accepted, an individual’s chronological age can be quite different from his or her biological age. Although there is no exact definition and measure for biological age, individuals of the same chronological age may vary in their “biological aging”, i.e. rate of declining integrity of multiple organ systems. Individuals who are aging more rapidly are typically less physically able, with greater cognitive decline and brain aging, lower self- reported health, and looking older^2^. Biological age is commonly used as a relative measure indicating whether the body is functioning better or worse than suggested by the chronological age^55,56^.

Given that both HGS and PWV are candidate markers of biological age, it would also be possible that there is not a unilateral or bilateral causal relationship between vascular aging and muscle mass decline, but they are associated simply because they measure the same trait. Our results and the above-mentioned findings of others suggest that low handgrip strength and arterial stiffness commonly go hand in hand.

Arterial stiffness has been established as an independent risk marker for cardiovascular disease, and well reflects the dissociation between chronological and biological age of large arteries. The concept of Early Vascular Ageing (EVA) has been suggested to identify individuals whose vessels prematurely show alterations typically found in more advanced chronological age^57^. These may be due to lifestyle (e.g. sedentary, no sports, smoking), high blood pressure, hypercholesterolemia, genetics, amongst others.

Likewise, handgrip strength declines with increasing age, and is widely used as simple and robust marker of biological age^58^. The PURE study suggested that simply measuring one’s handgrip strength could be a good way to assess biological age^18^.

PWV has not been widely integrated into the clinical routine yet, despite the intriguing additional information it provides. This study underlines that HGS is very well suited as a clinical risk marker. The test-retest reliability is excellent^59^. Since we may well infer from low HGS that PWV may be high—only 5.4% in the highest handgrip strength tertile had a high PWV > 11 m/s, compared to 39.4% in the lowest tertile—and measurement of HGS is done in a few seconds and inexpensive, it may be the ideal screening tool. In a similar vein, handgrip strength has been shown to correlate well with European System for Cardiac Operative Risk Evaluation (EuroSCORE) values^60^.

Of course, given the high interest in predicting biological age the most precisely possible, both markers HGS and PWV are candidates to be integrated in multimarker scores^61^.

Handgrip strength is inversely associated with PWV. While both are inexpensive and easy to measure, measurement of HGS is yet easier, quicker and less error-prone, and is highly reproducible. It is at least as effective in predicting adverse outcomes as PWV. Both seem to be suited for early identification of accelerated aging—even before chronic disease becomes established—offering opportunities for prevention. Therefore, adoption of HGS into clinical setting to identify or screen for individuals at greatest risk for future CV outcomes is appealing.

Furthermore, arterial stiffness may help explain the relationship between HGS and CVD. Not least it is conceivable that targeted interventions following a diagnosis of low HGS would be capable to improve arterial compliance.

### Strengths and limitations

In addition to the already mentioned aspects our study has several limitations.

We used aortal PWV (aPWV), which is the gold standard measure of arterial stiffness. Recently, criticism of cuff-based, or algorithm-based devices like the Mobil-O-Graph, which has been used in this study, has been expressed. It is being criticized that the estimation of PWV is largely dependent on age and blood pressure, thus arguably providing no extra information^62,63^.

On the other hand, there is good evidence that the Mobil-O-Graph provides similar results as devices noninvasively measuring cf-PWV, and shows good correlation with invasive aortic PWV^64–66^.

While we cannot prove or disprove the issues raised with the present work, we however, believe that the potential issues do not compromise our study.

Further limitations of our study include the observational nature and cross-sectional design of this study, which does not allow us to make conclusions on the causal role of muscular strength in arterial stiffness and cardiovascular disease, or vice versa. Conclusions on temporality are impossible, and residual confounding cannot be excluded. The observed association between handgrip strength and arterial stiffness may be causal, due to (unmeasured) confounding, or due to reverse-causation, i.e. reflecting early manifestation of underlying disease processes. Indeed, mendelian randomization analyses could not show that muscular strength is predictive for mortality risk, or cardiovascular events^8^.

Furthermore, the possibility of bias, both selection and information bias must be considered:

BASE-II is a convenience sample, whose participants were positively selected regarding education and health^31^. On the other hand, for the LipidCardio study, consecutive patients of a tertiary cardiology center, who all underwent coronary angiography, have been recruited^30^. Thus there is potential for selection bias, while the direction of bias is unpredictable.

As to the generalizability of the results, given the fact that the association was observed in two very different samples, we believe that the finding may well apply to the population of older adults with Caucasian ancestry in comparable living conditions in Central Europe.

## Conclusion

Handgrip strength was inversely associated with PWV, indicating that low handgrip strength and increased arterial stiffness go hand in hand. Arterial stiffness can possibly create the missing link between low handgrip strength and increased cardiovascular morbidity and mortality.

## Data Availability

Data can be requested from the steering committees of the Berlin Aging Study II and the LipidCardio Study. Further Information can be found under https://www.base2.mpg.de/en.
